# Multimodal dataset linking wide‐field calcium imaging to behavior changes in operant lever‐pull task in mice

**DOI:** 10.1038/s41597-025-05482-y

**Published:** 2025-07-29

**Authors:** Masashi Kondo, Keisuke Sehara, Rie Harukuni, Ryo Aoki, Shoya Sugimoto, Yasuhiro R. Tanaka, Masanori Matsuzaki, Ken Nakae

**Affiliations:** 1 Department of Physiology, Graduate School of Medicine, The University of Tokyo, Tokyo, Japan; 2 Brain Science Institute, Tamagawa University, Tokyo, Japan; 3 Graduate School of Brain Sciences, Tamagawa University, Tokyo, Japan; 4 Brain Functional Dynamics Collaboration Laboratory, RIKEN Center for Brain Science, Saitama, Japan; 5 Department of Biological Sciences, Graduate School of Science, The University of Tokyo, Tokyo, Japan; 6 International Research Center for Neurointelligence (WPI-IRCN), The University of Tokyo Institutes for Advanced Study, Tokyo, Japan; 7 Exploratory Research Center on Life and Living Systems, National Institutes of Natural Sciences, Aichi, Japan; 8 Graduate School of Engineering, University of Fukui, Fukui, Japan

**Keywords:** Neural circuits, Decision

## Abstract

The link between comprehensive behavioral measurements during a behavioral task and brain-wide neuronal activity is an essential strategy to better understand the brain dynamics underlying the emergence of behavior changes. To tackle this, we provide an extensive, multimodal dataset that includes 15 sessions spanning 2 weeks of motor skill learning, in which 25 mice were trained to pull a lever to obtain water rewards. Simultaneous high-speed videography captured body, facial, and eye movements, and environmental parameters were monitored. The dataset also features resting-state cortical activity and sensory-evoked responses, enhancing its utility for both learning-related and sensory-driven neural dynamics studies. Data are formatted in accordance with the Neurodata Without Borders (NWB) standard, ensuring compatibility with existing analysis tools and adherence to the FAIR principles (Findable, Accessible, Interoperable, Reusable). This resource enables in-depth investigations into the neural mechanisms underlying behavior and learning. The platform encourages collaborative research, supporting the exploration of rapid within-session learning effects, long-term behavioral adaptations, and neural circuit dynamics.

## Background & Summary

Understanding the relationship between neural activity and behavior remains a fundamental challenge in neuroscience, particularly in the context of learning. Operant conditioning paradigms^1,2^ have proven invaluable for studying behavioral adaptation and, together with conventional neurophysiological approaches, have uncovered fundamental neural correlates of behavioral adaptation^3–5^. Recent technical advances in wide-field calcium imaging^6–11^, coinciding with the emergence of deep-learning-based behavioral analyses^12–15^, offer unprecedented opportunities to correlate cortical activity patterns with precise behavioral quantification across large datasets.

Recent studies have highlighted that even seemingly task-unrelated movements, particularly subtle orofacial ones, can strongly modulate cortical activity in rodents^16,17^. These covert behaviors and their accompanying neural states can not only shape ongoing behavioral performance but may also influence longer-term outcomes of learning, underscoring the need for comprehensive datasets capable of capturing both overt and subtle expressions of behavior.

Here, we deposit a dataset in the BraiDyn-BC Database (https://braidyn-bc-database.netlify.app/), which builds on earlier studies^18,19^. Our dataset addresses the need for comprehensive, multimodal data in learning studies by combining wide-field calcium imaging of cortical activity with detailed behavioral measurements in mice during behavioral tasks under the head-fixed condition. Our experimental paradigm employed transgenic mice expressing GCaMP6s in excitatory neurons^20^, enabling fluorescent monitoring of neural activity across the dorsal cortex. In addition to behavioral sensors for task control and recording, three high-speed cameras (100 Hz) captured upper body, facial, and eye movements, while the atmospheric environment was continuously monitored. In total, 25 mice completed 15 training sessions of an operant lever-pull task, and we successfully collected data from 364 of 375 sessions, achieving a collection rate of 97%. The remaining 11 sessions could not be measured due to incomplete calcium imaging data.

The dataset includes not only task-related measurements but also resting-state cortical activity and sensory-evoked responses under anesthesia, enabling investigation of both task-dependent and baseline neural dynamics. Data are structured in accordance with the Neurodata Without Borders (NWB) format^21^, ensuring compatibility with existing analysis tools and adherence to the FAIR principles (Findable, Accessible, Interoperable, Reusable)^22^. By providing ready-to-use preprocessed data in addition to comprehensive raw data (~8 TB), we facilitate analyses ranging from rapid within-session learning dynamics to long-term behavioral adaptations and associated neural circuit changes. Considering the goal of mapping neural activity to behavior, our approach shares certain common objectives with other open, large-scale, cell-resolution datasets, including those from the International Brain Laboratory^23,24^ and Allen Brain Observatory^25^.

This resource aims to advance open neuroscience research by enabling investigations into the complex relationships between cortical activity, motor behavior, and environmental factors during learning^26^. The multimodal nature of our dataset supports diverse analytical approaches, from traditional behavioral metrics to advanced neural circuit analyses. Through open distribution of this comprehensive dataset, we seek to promote transparent, reproducible research and facilitate novel insights into the neural mechanisms underlying behavior changes.

## Methods

### Animals

Transgenic mice for wide-field imaging were obtained by crossing VGluT1-Cre mice (B6.Cg-*Slc17a7tm1.1(cre)Hze*/J, strain #: 037512, Jackson Laboratory)^27^ and Ai162 mice (B6.Cg-Igs7tm162.1(tetO-GCaMP6s,CAG-tTA2)Hze/J, strain #: 031562, Jackson Laboratory)^28^. All mice were allowed access to food and water *ad libitum* and were housed in a 12:12 h light-dark cycle (light cycle: 8 AM–8 PM). Both males (*n* = 11) and females (*n* = 14) were used for the experiments and were aged 17–31 weeks at the end of the experiments. For at least 3 days before surgery, mice were supplied and habituated with a carprofen-containing sweetened gel (3 g/day, MediGel Hazelnut or DietGel Boost, ClearH_2_O, ME, USA; 0.48 mg/g in gel, Remadile, Zoetis, NJ, USA), which was occasionally provided after surgery to decrease post-operative pain. All animal experiments were approved by the Institutional Animal Care and Use Committee of the University of Tokyo, Japan.

### Surgical procedures

Surgical procedures were performed according to our previous report (Kondo & Matsuzaki, 2021) with a small modification. Mice were anesthetized by intraperitoneal or intramuscular injection of a mixture of ketamine (74 mg/kg; Daiichi Sankyo Propharma, Tokyo, Japan) and xylazine (10 mg/kg; Beyer Pharma Japan, Osaka, Japan). After anesthesia, an eye ointment (0.3% w/v ofloxacin; Tarivid, Santen Pharmaceutical, Osaka, Japan) was used to prevent eye-drying and infection. During surgery, body temperature was maintained at 36–37 °C with a heating pad. The head of the mouse was sterilized with 70% ethanol, the hair was shaved, and the scalp was incised. After the skull was exposed, the soft tissue on the skull was removed. The temporal muscle was carefully detached from the cranium to improve the observation of temporal cortical areas. A custom head-plate (Misumi, Tokyo, Japan) was attached to the skull using dental resin cement (Estecem II, Tokuyama Dental, Tokyo, Japan). To prevent excitation light from directly entering through gaps between the head-plate and eyes of the mouse, the gaps were filled with a mixture of dental resin cement (Fuji lute BC; GC, Tokyo, Japan) and carbon powder (FUJIFILM Wako Pure Chemical, Osaka, Japan). To prevent drying of the skull surface, a thin layer of cyanoacrylate adhesive (Vetbond; 3 M, MN, USA) and dental resin cement (Super bond; Sun Medical, Shiga, Japan) were applied. After the curing of the dental resin layer, UV-curing optical adhesive (NOA81, Norland Products, NJ, USA) was applied several times. This enabled us to observe cortical activity longitudinally through an intact cranium. The optical adhesive-cured skull was covered with a silicone elastomer (Dent-silicone V, Shohu, Kyoto, Japan) to protect it from dust. An isotonic saline solution with 5% (w/v) glucose and the anti-inflammatory analgesic carprofen (5 mg/kg, Remadile) was injected intraperitoneally after all surgical procedures. The water control schedule was started after at least 3 days of recovery.

### Behavioral apparatus

All behavioral tasks and imaging were performed in darkness inside a dedicated sound-attenuating task box equipped with a lever unit, licking recorder, sound presentation system, and water reward feeder (O’hara & Co., Tokyo, Japan). We conducted all behavioral tasks and recorded analog voltages from the behavioral sensors with Labview (ver 2021, National Instruments, TX, USA). The atmosphere of the task box was monitored during training; temperature, humidity, atmospheric air pressure, and CO_2_ concentration were measured with electronic sensors (BME680, BOSHE Sensortec, Reutlingen, Germany; MH-Z19C, Zhengzhou Winsen Electronics Technology Co., Zhengzhou, China), which were connected to a microcontroller (Seeed XIAO RP2040, Seeed Technology Co., Shenzhen, China) by the I^2^C interface. Outputs of the atmospheric sensors were recorded on a PC at 20 Hz via a USB serial connection. The head-fixed mouse was placed on the body chamber. A lever and immobile pawrest were set in front of the right and left forelimbs, respectively^29,30^. The lever was movable but stabilized with a pair of permanent magnets at a base position. A force of 0.04 N was required for lever-pull initiation^30,31^. The lever could be pulled up to a distance of 4 mm, where it was physically blocked, and then could return to the base position without being pulled owing to the magnetic force. The position of the lever was recorded using a rotary encoder (MES-12-2000P, Microtech Laboratory, Kanagawa, Japan) equipped 80 mm away from the lever tip. The pulse outputs of the rotary encoder were counted with an NI-DAQ (USB-6229 or PCIe-6321, National Instruments, TX, USA), converted into the arc length, which was recorded with other analog data.

A lick spout was placed in front of the mouse, aligned with its mouth. Licking behavior was monitored by electrically detecting the contact between the tongue and lick spout, with the signals conveyed to an analog input of the NI-DAQ. All analog voltage inputs, frame synchronization pulses, and values from the environmental sensor were recorded at a sampling rate of 5 kHz. Sound cues (10 kHz sinusoidal tone, 70 dB sound pressure level, SPL) were presented through a speaker (FT28D, Fostex, Tokyo, Japan) on the left side, 25 cm from the animal. The water reward (4 μL/drop) was fed with a micro pump unit and released from the lick spout mentioned above. Body movement was recorded with a load cell with a rated capacity of 100 g. The displacement detected by the load cell was processed with an instrumental amplifier (LT1167, Analog Devices, MA, USA) into an analog voltage and transmitted to an analog input of the NI-DAQ. This analog voltage was not calibrated with an actual weight, and thus the unit was arbitrary. Visual stimuli were emitted from the tip of a φ5 mm stainless steel tube, attached with a red LED on its opposite side, and vibro-tactile stimuli were generated as a vibration (linear vibration actuator, LD14-002, Nidec, Kyoto, Japan) or an air puff (0.1 MPa) on the whisker pad. The transistor-transistor-logic (TTL) signals controlling reward delivery or sensory stimuli were recorded as an analog voltage with NI-DAQ.

### Behavioral task

Our task schedule spanned approximately 1 month. First, to facilitate the acclimatization of mice to the environment, we conducted 3–8 days of pre-training (see below). On the last pre-training day, we conducted the first resting-state recording session after the pre-training (recording day 0). On the following days, we conducted one task training session per day, with occasional training breaks of 2–3 days without any handling (*e.g*., over weekends; 3.1 ± 0.6 (SD) breaks throughout training; 2.3 ± 0.7 days/break; *n* = 25 mice). Resting-state recording sessions were also conducted after task training sessions on recording days 1, 7, and 15. Of note, for one animal, the mid-training resting-state session was performed on recording day 8 instead of day 7 (Table 1). On recording day 16, *i.e*., 1–6 days after the 15^th^ recording day, the sensory-mapping session was held. The entire recording period (recording days 1–16) spanned 24.3 ± 3.2 calendar days.

**Table 1:** Summary of the numbers of different types of sessions reported in this dataset.

	Base recording		Seesions with videos			
	Animals	Session	Upper body	Face	Eye	All views
**Task**	25/25	364/375	357/364	354/364	356/364	353/364
**Resting state(day 0)**	25/25	25/25	20/25	20/25	20/25	20/25
**Resting state (day 1)**	25/25	25/25	25/25	25/25	25/25	25/25
**Resting state (day 7)**	24/25	24/24	23/24	23/24	23/24	23/24
**Resting state (day 8)**	1/25	1/1	1/1	1/1	1/1	1/1
**Resting state (day 15)**	25/25	23/25	23/23	23/23	23/23	23/23
**Sensory mapping**	25/25	33/33	—	—	—	—

Of all the sessions performed and recorded, only those with associated imaging data are deposited here. The denominators in the “Animals” column indicate the total number of animals trained for this dataset (*n* = 25). The denominators in the “Session” column indicate the total number of sessions performed in the corresponding category. The denominators in the other columns correspond to the numbers of sessions during which imaging data was successfully recorded. For eight of the animals, two sensory-mapping sessions were performed. Of note, some sessions experienced failures when recording videos from one or more views. Videos were not recorded during sensory-mapping sessions.

#### Tone-triggered lever-pull task

The lever-pull task was performed for 30 min per session each day. A lever pull was defined as the epoch during which the mouse continuously kept the lever pulled for >1 mm from its base position (the maximum pull limit was 4 mm). A trial started with presentation of the sound cue (10 kHz pure tone, 70 dB SPL, 200 ms). A trial was considered successful if the mouse pulled the lever for longer than a defined duration of time (*T*_pull_) within 1 s from the sound cue onset. For each success, a water reward (4 μL) was automatically delivered from the lick spout.

We ensured that the mouse could try pulling the lever only once during the 1 s cued period, by monitoring the lever position online. Even before the 1 s had passed for the cued period, once the lever was put into the pulled state, and returned to the non-pulled state before the required duration *T*_pull_ had elapsed, we considered the trial to be a failure, and aborted the trial. Conversely, if the mouse did not put the lever into the pulled state at all during the cued period, we considered the trial to be a miss.

The inter-trial interval between 3–4 s was randomly set after each trial, and if a mouse pulled the lever during the inter-trial interval, the interval was extended by another 3–4 s until the next sound cue. The required lever-pull time, *T*_pull_, was adjusted according to the behavioral performance. The initial *T*_pull_ in the first session was set to 1 ms. It was extended by 50 ms when there was an 80% success rate in the previous 20 trials, up to the maximum duration of 400 ms. We set a refractory period of 20 trials for these extensions, ensuring that each extension occurred at least 20 trials apart. We defined the *T*_pull_final_ of a session as the *T*_pull_ at the end of the session, and the initial *T*_pull_ in the next session was set to *T*_pull_final_ of the previous session minus 100 ms. *T*_pull_ was never shortened within the same session.

#### Resting-state recording

On recording days 1, 7, and 15, resting-state recording was conducted after sufficient intake of water after finishing the lever-pull task. Of note, for one animal, the mid-training resting-state session was performed on recording day 8 instead of day 7 (Table 1). The recording duration was 10 min. On day 0, the resting-state recording was performed after the pre-training.

#### Sensory mapping

After 15 lever-pull task sessions, mice were allowed to drink water freely in their home cage. After 1–6 days, a sensory-mapping session was performed. The recording time was 15 min under anesthesia with a mixture of fentanyl (0.05 mg/kg; Daiichi Sankyo Co., Tokyo, Japan), midazolam (5.0 mg/kg; Sandoz, Tokyo, Japan), and medetomidine (0.5 mg/kg; Zenoaq, Fukushima, Japan)^32^. Mice were stimulated visually with a red LED (1 s duration, 0.5 duty cycle, 10 Hz), tactilely with vibration on the right whisker pad (~150 Hz; occasionally, with air puff to the whisker pad), and auditorily with white noise (1 s, ~70 dB SPL) from the speaker. These stimuli were presented in a fixed order (visual, tactile, auditory), and the inter-stimulus intervals were randomly set to 10–15 s in each trial. After the session, animals were recovered with antagonistic drugs: flumazenil (0.5 mg/kg; Nippon Chemiphar, Tokyo, Japan), atipamezole (2.5 mg/kg; Zenoaq, Fukushima, Japan), and naloxone (1.2 mg/kg; Alfresa Pharma, Osaka, Japan).

#### Water control and pre-training

At least 2 days before starting training sessions, the water in the home cage was replaced with water containing 2% citric acid^33^. This replacement mildly suppressed the daily water intake of mice. During the period of daily training, mice typically obtained the necessary water (>1 mL) in training sessions, and they had unrestricted access to standard pellets in their home cages. When a training break lasted ≥2 days (*e.g*., over weekends), water containing 2% citric acid was placed in the home cage. These bottles were removed the morning of the next training day.

For acclimatization to the head-fixed condition in the task box, mice were pre-trained. In the pre-training, mice were head-fixed and received water rewards in a similar manner as in the lever-pull task, except they obtained the reward by licking the lick spout rather than pulling the lever. Pre-training was conducted 3–8 times, and the numbers of pre-training sessions were included in the metadata. We noticed that some mice gradually lost weight with time even if the mice received daily water intake in the behavioral session. Therefore, to maintain the body weights of mice at approximately >80% of the body weight before water restriction, we provided mice with additional water and high-caloric food (Calorie-Mate fruit flavor, Otsuka Pharmaceutical Co., Tokyo, Japan) when necessary after the behavioral session.

### Acquisition of wide-field one-photon images

For wide-field one-photon calcium imaging, a wide-field tandem-lens macroscope (THT mesoscope, Brain Vision, Tokyo, Japan), equipped with an objective lens (PLAN APO 1×, #10450028, Leica Microsystems, Wetzlar, Germany) and imaging lens (F2.0, focal length 135 mm, Samyang, Seoul, Republic of Korea), was used. Images were acquired with a CMOS camera (ORCA-Fusion BT, C14440-20UP, Hamamatsu Photonics, Shizuoka, Japan) and HCImage software (ver 5.0.2.2, Hamamatsu Photonics). Single images consisting of 588 × 588 pixels were captured at 60 Hz. We alternately illuminated two excitation LEDs with different wavelengths (405 nm and 470 nm; M405LP1 and M470L5, Thorlabs, NJ, USA) and used band-pass emission filters (FBH405-10 for 405 nm and FBH470-10 for 470 nm, Thorlabs). These lights were combined with a dichroic mirror (DMLP425R, Thorlabs) and delivered to the macroscope through a liquid light guide (Ø5 mm Core) and collimator (LLG5-6H and COP1-A, Thorlabs). The collimated light was passed through the 3D-printed field stop (the geometry was designed for the inner space of the head-plate) and the condenser lens (plano convex lens, f = 150 mm, LA1417, Thorlabs), and a dichroic beam splitter (FF484-FDi01, Semrock; IDEX Health & Science, NY, USA) then projected to the sample specimen. For calcium imaging, a 3D-printed light shield was placed on the head-plate to prevent direct illumination of excitation light to the eyes of the mouse. The total power of the excitation lights (blue and violet) was set at ~10 mW, and fluorescent signal degradation across imaging sessions was not observed. Fluorescent emission signal from the sample was collected with the objective and passed through the dichroic band-pass filter (FF01-536/40, Semrock; IDEX Health & Science) and imaging lens, and then projected to the CMOS camera. Two sequential images obtained by two different excitation lights are analyzed as a pair (see below), and thus 30 Hz is considered the effective sampling rate. LED illumination and image acquisition timing signals were recorded using the same DAQ device (USB-6229 or PCIe-6321) used for the task. We imaged 108000 frames (30 min) in each task session, 36000 frames in each resting-state session (10 min on recording days 1, 7, and 15), and 54000 frames in each sensory-mapping session (recording day 16).

### Acquisition of high-speed videography

Three machine vision cameras (acA1440-220um, Basler, Ahrensburg, Germany) recorded videos of the upper body, right side of the face, and right eye at 100 Hz. All recording and controlling machine vision cameras were conducted with Pylon viewer (ver 7.2.1.25747, Basler, Ahrensburg, Germany). Pulses (5 V, 100 Hz, duty rate of 0.5) from the DAQ device were used for synchronization of the frame acquisition across cameras and were recorded as an analog voltage. The band-pass IR cut filter (center 850 nm, 25 nm FWHM, Edmund Optics, NJ, USA) was placed in front of the imaging lens of each camera. The videos were saved as MP4 files. LED arrays (OSI3CA5111A, OptoSupply, Hong Kong; consisting of 850 nm 64 LEDs) at the back of each camera were used as light sources. In addition, a 910 nm LED was lit in front of the mouse. The brightness of the 910 nm LED was modulated in each session, so that the pupil diameter would dilate moderately during imaging, without any adjustments during individual sessions.

### Data processing

Behavioral data processing (filtering, event detection, lick-rate calculation, and temporal down-sampling to synchronize calcium imaging data) and imaging data processing (spatial down-sampling, interleaved image separation of two excitation wavelengths, and motion correction) were conducted with MATLAB software suite (2022b; Mathworks, MA, USA) as described in the following subsections. All other data processing was conducted in a Python environment (conda version 24.3.0; Python version 3.10.14) with appropriate software packages (NumPy version 1.24.3; SciPy 1.14.0; scikit-learn version 1.5.1; Pandas version 2.2.2; h5py version 3.11.0; PyNWB version 2.8.1; OpenCV python binding, version 4.10.0). The source codes of all processing workflows are deposited at https://github.com/BraiDyn-BC/bdbc-data-pipeline.

#### Event detection from behavioral sensors and synchronization for calcium imaging data

Each task event (onsets of tone cue, reward, lever pulls, and sensory stimuli in the sensory-mapping experiment) was detected by an appropriate threshold. The motion sensor output was processed by applying a 10 Hz cut-off low-pass filter and then subtracting its time average. A 0-1 function indicating the voltage rise time points in the licking sensor was convolved with a 0.5 s exponential kernel to yield the lick rate. Raw data sampled at 5 kHz were embedded into the 30 Hz imaging frames as follows: any sound cue, reward delivery, or sensory stimulus occurring between the end of one frame and the beginning of the next was assigned to the earlier frame during down-sampling. Lever position, lick rate, and environmental sensor values were averaged over the interval from the onset of a frame to the onset of the following frame to generate the values for the preceding frame.

#### Image processing, registration to standard brain atlas, hemodynamics correction, and calcium activity extraction from wide-field one-photon imaging data

Images were down-sampled into 288 × 288 pixels and divided into two image stacks according to the recorded LED pulse timings. One image stack contained images acquired with blue light excitation (*I*_B_), and the other contained images acquired with violet light excitation (*I*_V_). The displacement of each frame of *I*_B_ was estimated with the NoRMCorre-based rigid frame registration^34^ using the time-averaged image of *I*_B_ as a reference. The calculated displacement vector was applied to both *I*_B_ and *I*_V_ stacks, and these motion-corrected *I*_B_ and *I*_V_ stacks were treated as raw data in the datasets.

To draw borders of neocortical areas in our imaging data, we first prepared a template frame for each animal, and aligned the Allen common coordinate framework (Allen CCF)^35^ with this template frame using an approach based on MesoNet^36^ (the ks-mesoscaler, ks-affine2d, ks-affine-aligner, and bdbc-atlas-registration libraries). The animal-by-animal template frames were computed by alignment of the session-average frames with each other; for each session, we first computed the mean of the 470 nm excitation frames over time. We then selected one of these session-mean images as the animal-representative image, and estimated affine transformation matrices for conversion to this representative image from the other session-mean images, based on the keypoints detected using the Oriented FAST and rotated BRIEF (ORB) descriptor^37^ of the Python OpenCV library. Using these affine matrices, individual session standard-deviation images over time were warped (using the ks-affine2d library) and then averaged to obtain the animal-by-animal template frames. The landmark-inference DeepLabCut^12^ network from MesoNet^36,38^ was used to estimate the nine landmarks on the skull of the animal-by-animal template frames. To estimate the affine transformation from Allen CCF to each template frame, the landmarks being estimated with a likelihood above 0.85 were aligned with those defined on the Allen CCF (provided by the authors of MesoNet; https://github.com/bf777/MesoNet/tree/master/mesonet/atlases/atlas). Finally, based on the two affine matrices (*i.e*., the atlas to the animal template and the animal template to the session mean), we computed the transformation from Allen CCF to each session-mean image (using the ks-affine2d library). The region of interest (ROI) masks, provided by the MesoNet package, were transformed using this final affine matrix to generate corresponding binary masks of neocortical areas. The signal intensity was averaged across ROIs into a time series of signals.

To enhance the usability of our dataset, we provided ROI signals with hemodynamics correction using a method similar to that conventionally used by others^17,39,40^. Fluorescent signals obtained with blue-excitation light (*F*_B_) mainly contained calcium-dependent signal changes but with small contamination of hemodynamic fluctuations (ratio of oxy- and deoxyhemoglobin and the total volume of blood vessels and arteries)^41^. We therefore used fluorescence obtained with violet-excitation light (*F*_V_) as an instantaneous reference of calcium-independent fluorescence fluctuations, as this wavelength is near the isosbestic point of GCaMP. We thus corrected *F*_b_ based on *F*_v_ using the following procedure. First, ratiometric signals were calculated as ∆*F*_B_/*F*_B_ = (*F*_B_ − *F*_B0_) / *F*_B0_ and ∆*F*_V_/*F*_V_ = (*F*_V_ − *F*_V0_) / *F*_V0_, with *F*_B0_ and *F*_V0_ representing the median of *F*_B_ and *F*_V_ over time, respectively. The two time series, ∆*F*_B_/*F*_B_ and ∆*F*_V_/*F*_V_, were then band-pass-filtered to obtain (∆*F*_B_/*F*_B_)_filtered_ and (∆*F*_V_/*F*_V_)_filtered_, with removed high-frequency acquisition noise and low-frequency baseline drifts. For this, the “filtfilt” function of SciPy (version 1.14.0) was applied in combination with the fifth-order 0.01–10 Hz band-pass butterworth filter, with a frequency consistent with previous studies^39,40^. Finally, linear regression was performed to predict (∆*F*_B_/*F*_B_)_filtered_ based on (∆*F*_V_/*F*_V_)_filtered_; this was used to estimate hemodynamics effects: (∆*F*_B_/*F*_B_)_hemo_ = *A* × (∆*F*_V_/*F*_V_)_filtered_ + *b*, with *A* and *b* representing the slope and bias term, respectively. The hemodynamics-corrected calcium signal was computed as the residuals of the regression, *i.e*., (∆*F*_B_/*F*_B_)_corrected_ = (∆*F*_B_/*F*_B_)_filtered_ − (∆*F*_B_/*F*_B_)_hemo_.

#### Motion estimation of body parts from high-speed videography

We used DeepLabCut version 2.3.10^12^ to estimate the keypoints representing body-part positions in the behavioral videos (Fig. 2; Table 2). The strategy for image extraction from videos for neural-network model preparation is described in Fig. S3, with the number of images used summarized in Table 3 (see also Fig. 6). To improve the generalization of some models, we additionally used some videos from different experiments that were obtained in the same behavioral rig for the initial training iterations. For later training iterations, training and testing of the models were performed in an incremental manner; we verified the performance of the model in tracking a set of videos, before extracting frames from another set of videos. Frames were not extracted from the videos if the tracking performance of the model was considered satisfactory. Prior to each training iteration, an image augmentation step was inserted using the “imgaug” Python package (version 0.4.0) to produce 10–20 augmented images based on each of the annotated images to be used for training and testing. The coordinates were set so that the x-axis increases as points move to the right, and the y-axis increases as points move downward. Keypoint estimation by DeepLabCut was inherently performed even when the body part in question was absent. Therefore, in our published data, we shared not only the raw estimations from DeepLabCut but also the likelihood of the estimation for the potential filtering purposes. To estimate the size and position of the pupil, the set of circumferential points along the pupil boundary was fitted with an ellipse on a frame-by-frame basis. The center position and the length of the major axis of the fitted ellipse were defined as the position and the diameter of the pupil, respectively.

**Table 2:** Summary of keypoints (Fig. 2b–d).

Keypoints in videos of the upper body		
1	Spout, tip	The tip of the lick spout
2	Forepaw, right	The center of the right forepaw
3	Forepaw, left	The center of the left forepaw
4	Lever, tip	The tip of the lever
5	Pawrest, tip	The tip of the pawrest
Keypoints in videos of the right side of the face		
1	Spout, tip	The tip of the lick spout
2	Tongue, tip	The tip of the tongue in the midline
3	Jaw, lower	The tip of the lower jaw in the midline
4	Nose, tip	The nose tip in the midline
5	Nose, right	The most lateral point of the right nostril wall
6	Forepaw, right	The center of the right forepaw
7	Forepaw, left	The center of the left forepaw
8	Nose, bottom	The most ventral point of the nose in the midline
9	Nose, root	The nose root in the midline
10	Eye, medial	The inner corner of the right eye
11	Eye, lateral	The outer corner of the right eye
12	Ear, root	The most anterodorsal tip of the right ear root
13	Ear, tip	The most lateral point of the right ear helix
14	Ear, lateral	The ventrolateral midpoint of the right ear helix
Keypoints in videos of the right eye		
1	Pupil borders	Thirty circumferential points on the pupil
2	Eye, inner corner	The medial corner of the right eye
3	Eye, outer corner	The lateral corner of the right eye
4	Pupil, center*	The center of the right pupil

*This keypoint was not directly estimated by DeepLabCut but through fitting of an ellipse to the pupil edges (see Methods).

**Table 3:** Summary of training and validation of the DeepLabCut models.

	Training and testing				Validation		
	Extracted			Annotated	Annotated		
	Animals	Sessions	Images		Animals	Sessions	Images
**Upper body**	19 + 4	35 + 8	1360 + 40	1399	25	96	192
**Face**	6 + 0	12 + 0	240 + 0	240	25	96	192
**Eye**	23 + 4	39 + 8	720 + 40	676	25	91	180

Training of the models was performed incrementally, through checking video files one-by-one. The numbers of animals, sessions, and images for training and testing (*left*) corresponds to the numbers from the videos of the current dataset, whereas those for validation (*right*) indicate the numbers from different experiments in the same behavioral rig, with the same video-camera configurations. For more details of our testing and validation strategy, see Fig. S3. For the eye model, in particular, discrepancy between the numbers of extracted and annotated frames occurs due to extraction of images when the animals were blinking.

For the resampling of the positional time series from the videography frame rate (100 Hz) to the effective imaging frame rate (30 Hz), we first up-sampled the series to the NI-DAQ sampling rate (5 kHz) and then down-sampled them to 30 Hz. During up-sampling, we dropped the inferred keypoint positions whose likelihood was < 0.2 and plugged the remaining values into the durations of their corresponding video pulses in the 5 kHz NI-DAQ recording. Inter-pulse interpolation was then performed only when the two neighboring pulses contained valid values. For the imaging pulses, two neighboring LED pulses (one violet and one blue) were merged to obtain the 30 Hz pulses. The mean over the duration of each of these pulses was computed to obtain the series down-sampled to the imaging frame rate.

## Data Records

The full dataset files are available at the DANDI Archive^42^, https://dandiarchive.org/dandiset/001425/ (10.48324/dandi.001425/0.250705.0947). The preprocessed dataset files in NWB format, without raw imaging data, are also available at the GIN^43^ https://gin.g-node.org/BraiDyn-BC/Kondo2025_CuedLeverPullNWB (10.12751/g-node.zbh16l).

We also provide online tutorials of how our dataset may be read and analyzed, as Google Colab notebooks, at https://drive.google.com/drive/folders/1QciTJd3tXkEGhz6782czB2dEO3fafm8M.

Our dataset includes data from the 15 behavioral task sessions and four resting-state recording sessions. Each experimental condition and session provides data in both raw and processed forms (see below). For each session, the dataset comprises one NWB file, two TIFF files, and three MP4 files. This data packaging into the NWB format was conducted in a Python environment.

### NWB Files

The NWB file contains both raw and processed data as well as associated metadata (Table 4). Processed data include:

1. Imaging: calcium activity data segmented into anatomical regions defined by the Allen CCF (22 ROIs/hemisphere).
2. Estimated positions of body parts: body part and pupil coordinates extracted by DeepLabCut from the three video recordings. We provide the data at the original video frame rate as well as those down-sampled to the imaging frame rate.
3. Behavioral data: the data was synchronized and processed according to the imaging frame timestamps. The NWB file also contains the corresponding raw behavioral data channels.

**Table 4:** Summary of the behavioral and sensor data stored in each NWB file along with the imaging and video data.

Name	Raw	DS	Unit	Type	Description
tone	○	○^(*)^	V	Digital (TTL)	Pulses representing when the auditory cue (10 kHz pure tone) is on.
lever	○	○	mm	Analog	The distance of the lever from its baseline position.
reward	○	○^(#)^	V	Digital (TTL)	The pulses used for reward delivery.
lick	○	○^(*)^	V	Digital (TTL)	The output of the lick sensor indicating when the animal licked the spout.
lick_rate	—	○	Hz	Analog	The lick rate calculated by applying an exponential kernel to the lick-event onset series.
motion	○	○	a.u.	Analog	The output of the load-cell motion sensor.
pull_duration	○	—	ms	Integer	The minimum duration for the animal to keep pulling the lever during the trial at a given time point.
state_lever	○	○	NA	Boolean	The binary status indicating whether the lever is in the pulled state.
state_task	○	○	NA	Integer	The state of the task: waiting phase (0), cued phase (1), or reward phase (2).
air_pressure	○	○	hPa	Analog	The atmospheric air pressure level around the behavioral setup.
CO2_level	○	○	ppm	Analog	The atmospheric carbon dioxide level around the behavioral setup.
humidity	○	○	%	Analog	The atmospheric humidity around the behavioral setup.
room_temp	○	○	°C	Analog	The atmospheric temperature around the behavioral setup.
LED_B	○	—	V	Digital (TTL)	The pulses representing when the blue (470 nm) LED is on.
LED_V	○	—	V	Digital (TTL)	The pulses representing when the violet (405 nm) LED is on.
img_acquisition	○	—	V	Digital (CMOS)	Pulses from the camera for calcium imaging, each of which corresponds to the timing when a single frame becomes ready.
video_trig	○	—	V	Digital (TTL)	The pulses representing when video-frame acquisition should be started.

DS, down-sampled data. *For these signal types, binary values (indicating the occurrence and duration of individual events) are stored instead of the original TTL signals. ^#^For reward-delivery pulses, binary values (indicating the onsets of individual reward-delivery events) are stored instead of the original TTL signals.

The NWB file includes the session_description identifier, timestamps_reference_time (timestamp reference or session start time), general metadata (session_id, experimenter, lab, institution, as well as subject information such as age, genotype, sex, species, subject_id, weight, date_of_birth, strain), and device information. All data channels are stored under acquisition (for raw data) and within analysis or processing (for processed data). The analysis group includes atlas_to_data_transform, an affine transformation matrix mapping the Allen CCF atlas (512 × 512 pixels) to the acquired imaging data (288 × 288 pixels).

In processing/behavior/data_interfaces, DeepLabCut-extracted keypoints are stored (eye_video_keypoints, face_video_keypoints, body_video_keypoints), along with eye_position (center_x and center_y) and pupil_tracking. The downsampled/data_interfaces/trials group contains trial information synchronized with imaging, as well as the down-sampled sensor data (CO2_level, air_pressure, humidity, lever, lick rate, motion, reward, room_temp, state_lever, state_task, tone).

### Imaging raw data

The full dataset files contain wide-field one-photon imaging data of dorsal cortical calcium activity in mice expressing Ca^2+^ probes in excitatory cortical neurons. During recording, blue and violet-excitation lights were alternately applied at 60 Hz to measure both calcium-dependent and calcium-independent fluorescence signals. After data acquisition, frames were separated by excitation wavelength, motion-corrected, and spatially down-sampled. The resulting data are stored as NWB OnePhotonSeries data entries of 288 × 288 pixels at frames of recording duration [s] × 30 Hz.

Please note that the dataset files on GIN repository^43^ are intended to be lightweight, so the NWB files do not contain the raw imaging data entries.

### Videography raw data (MP4 Files)

1. Body Camera Movie: sub-(animal)_ses-(date)-(task/resting-state)-day(day#)_body-camera.mp4
2. Face Camera Movie: sub-(animal)_ses-(date)-(task/resting-state)-day(day#)_face-camera.mp4
3. Eye Camera Movie: sub-(animal)_ses-(date)-(task/resting-state)-day(day#)_eye-camera.mp4

These MP4 files capture the mouse’s upper body, right facial region, and right eye at 100 Hz using three cameras. The cameras are synchronized based on a 5 V and 100 Hz pulse train (50% duty cycle) from a D/A converter. This pulse train is recorded as analog data and can be used to synchronize the videos with both behavioral and calcium activity data.

## Technical Validation

**Fig. 1:**
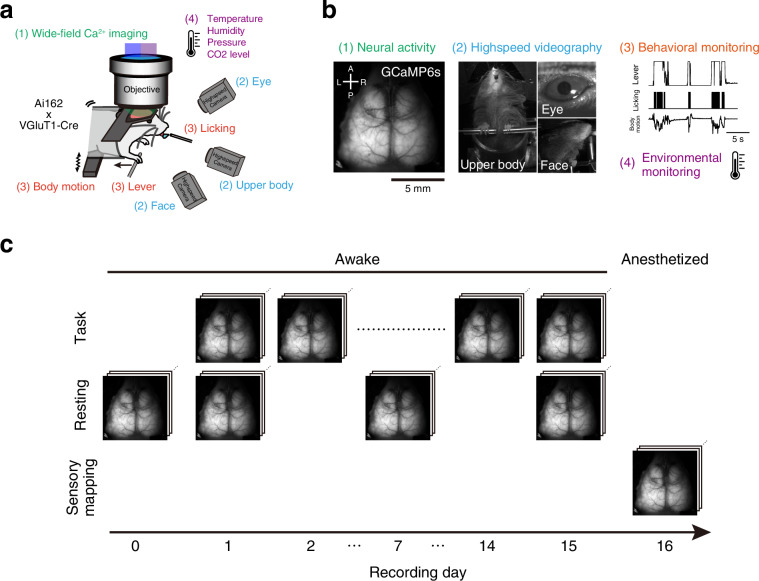
Experimental setup and timeline. (**a,b**) Schematic representations of the experimental setup and data structure. The text colors of the setup elements in (**a**) correspond to the text colors of the four components of the raw data from typical sessions in (**b**). (**c**) Timeline of the operant task and other experiments (see Methods for details).

**Fig. 2:**
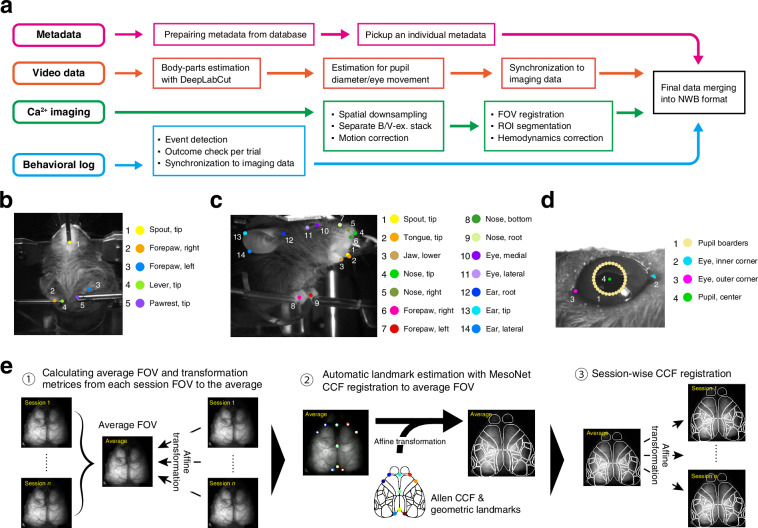
The processing pipeline of the dataset, estimated body parts by DeepLabCut, and FOV registration across animals and sessions. (**a**) The analytical pipeline. FOV, field of view; ROI, region of interest. (**b**–**d**) Keypoints in videos of the upper body (**b**), right side of the face (**c**), and right eye (**d**), detected by DeepLabCut. These keypoints are also summarized in Table 2. (**e**) Schematic illustration of atlas registration to Allen CCF using MesoNet and affine transformations (see Methods).

We provided a dataset containing 1) cortical calcium activity obtained with one-photon wide-field imaging, 2) video-tracked body-part movements, 3) sensor-monitored behavioral measurements, and 4) atmospheric environmental parameters during the operant lever-pull task (Fig. 1a, b; Table 1). The dataset includes not only 15 task training sessions but also four resting-state recording sessions and one sensory-mapping session per animal (Fig. 1c). The data were processed and integrated into session-wise NWB files by the newly developed analysis pipeline, which is available in a public GitHub repository (Fig. 2). We included both raw and processed data in the dataset, aiming to enable users to quickly utilize data for their purpose and, at the same time, to ensure that they can analyze the data from scratch and even beyond our scope. We validated the structure of the data of each session by a test code, which is also provided in the GitHub repository.

**Fig. 3:**
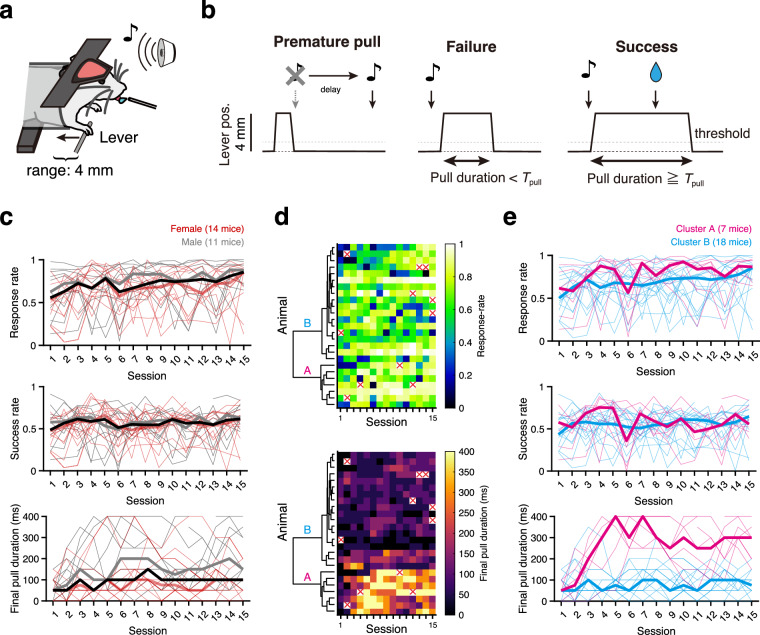
Behavioral performance in task training sessions. (**a,b**) Schematic illustrations of the lever-pull task for head-fixed mice. In response to the sound cue, mice should pull the lever and hold it for a specified duration to receive a water reward from the lick spout (**a**). Premature pulls before the sound cue caused an additional delay in sound cue presentation. If the lever was pulled within 1 s after the sound cue, and the position exceeded 1 mm (threshold) for the predetermined duration (*T*_pull_; see Methods for details), the lever pull was considered successful, and a water reward was provided. An insufficient pull or no response within 1 s after the sound cue was considered a failure. (**c**) Response rate (number of valid pulls/number of sound cues; top panel), success rate (number of successful pulls/number of sound cues; middle panel), and final pull duration (*T*_pull_final_; see Methods for details; bottom panel) across sessions. Thin lines show performances of individual female (red) and male (gray) animals, whereas thick lines represent medians across the population (red, female; gray, male; black, all). (**d**) Color-coded response rates and *T*_pull_final_ across sessions. In some sessions, data were missing for various reasons (red crosses; 11/375 = 0.29%). The result of hierarchical clustering (Ward’s method) based on the chronological change in *T*_pull_final_ is shown alongside. (**e**) Behavioral metrics of individuals in cluster A (*n* = 7; magenta) and B (*n* = 18; cyan) identified in (**d**). These plots are shown similarly as in (**c**).

During the operant lever-pull task (Fig. 3a,b), mice (14 females, 11 males) showed various behavioral changes. The response rate to the sound cue tended to increase overall (with the median of ~0.55 in task training session 1 increasing to ~0.85 in task training session 15), whereas the success rate was constant as a whole (Fig. 3c). To demonstrate inherent animal-to-animal variability in behavioral changes, we focused on how the animals pulled the lever during task training sessions. Provided that the animals do not know for how long they must pull the lever, one reasonable strategy for efficiently obtaining rewards would be to pull the lever for as long as possible. We thus reasoned that the examination of *T*_pull_final_ over the 15 task training sessions would reflect how each animal successfully adopted this strategy, and therefore would reveal the animal-to-animal variability in behavioral strategies as the training proceeded. In our dataset, there were two distinct trends of change in *T*_pull_final_ over the course of the task training sessions (Fig. 3c,d). Hierarchical clustering revealed that mice could be divided into two clusters in terms of *T*_pull_final_ (Fig. 3d). Mice from cluster A showed high success rates in the early sessions (~5) leading to high *T*_pull_final_, which were often maintained even in the late sessions. Mice from cluster B rarely achieved a high *T*_pull_final_, whereas they accomplished a response rate and success rate similar to cluster A mice in the late sessions (Fig. 3e, Fig. S1). These visualizations validate that the present dataset captures various behavioral changes during task training sessions that could help users of this dataset unfold the complex interactions between changes in environment, dynamics of behavioral strategy, and individuality of animals^44–47^. Of the 375 task training sessions conducted, we successfully collected data from 364 sessions (Fig. 3d; Table 1) and completed data collection for all 15 sessions in 16 of 25 animals.

**Fig. 4:**
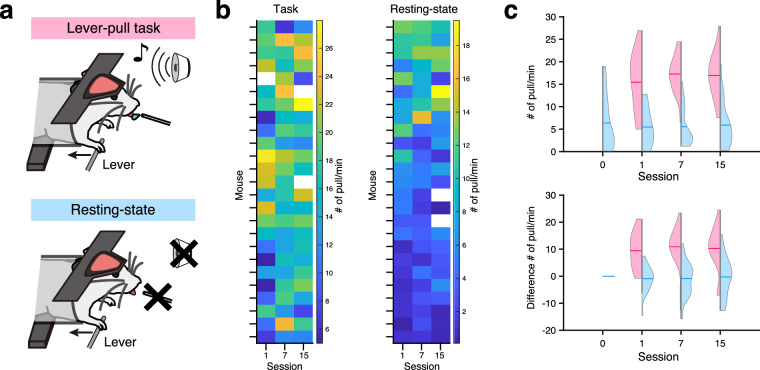
Differences in lever-pull frequency during the resting-state and task conditions. (**a**) Schematic illustrations of behavioral recordings during the resting-state and task sessions. In the task session (top), mice must pull the lever to earn the water rewards in response to the tone cue (see Methods). In the resting condition (bottom), tone cues and water rewards were not presented; however, mice could freely pull the lever. (**b**) The number of lever pulls/min is shown in each individual animal. Left and right panels correspond to the task and resting-state conditions, respectively. Animals are sorted in descending order by a sum of the numbers of lever pulls/min in sessions 1, 7, and 15 of the resting-state session. Transparent tiles (*e.g*., the 14^th^ and 16^th^ from the top in the most right-hand column of the resting-state) correspond to invalid data points due to missing data. (**c**) Violin plots of lever pulls/min during the resting-state (cyan) and task (magenta) conditions (top). If the lever was pulled continuously at 100 ms, the lever pull was considered as “pulled.” Cyan and magenta horizontal bars in each plot indicate the mean value. Violin plots of the differences in the numbers of lever pulls/min from resting-state recording day 0 (bottom).

To help users elucidate the potential impact of behavioral training on animals’ basal activity levels, we also included in our dataset the neural and behavioral recordings of the same set of animals during resting-state sessions. During these sessions, animals were awake and head-fixed in the setup without performing any explicit behavioral task. We compared lever-pull behavior, regardless of whether it was cue-dependent or not, between the operant-task and resting-state sessions, recorded on the same days (Fig. 4a). In general, the number of pulls/min tended to be larger during the operant-task sessions than during the resting-state sessions (Fig. 4b,c). Although we observed no changes in the number of pulls during the resting-state sessions over the period of operant-conditioning task training at the population level, we did observe changes at the individual level (Fig. 4b). During resting-state sessions, mice either tended to pull more frequently after their first day of task training, pulled more frequently later on during task training, or did not show any noticeable differences over the training period. This animal-to-animal variability may reflect the variability in how the mice comprehended the behavioral context.

**Fig. 5:**
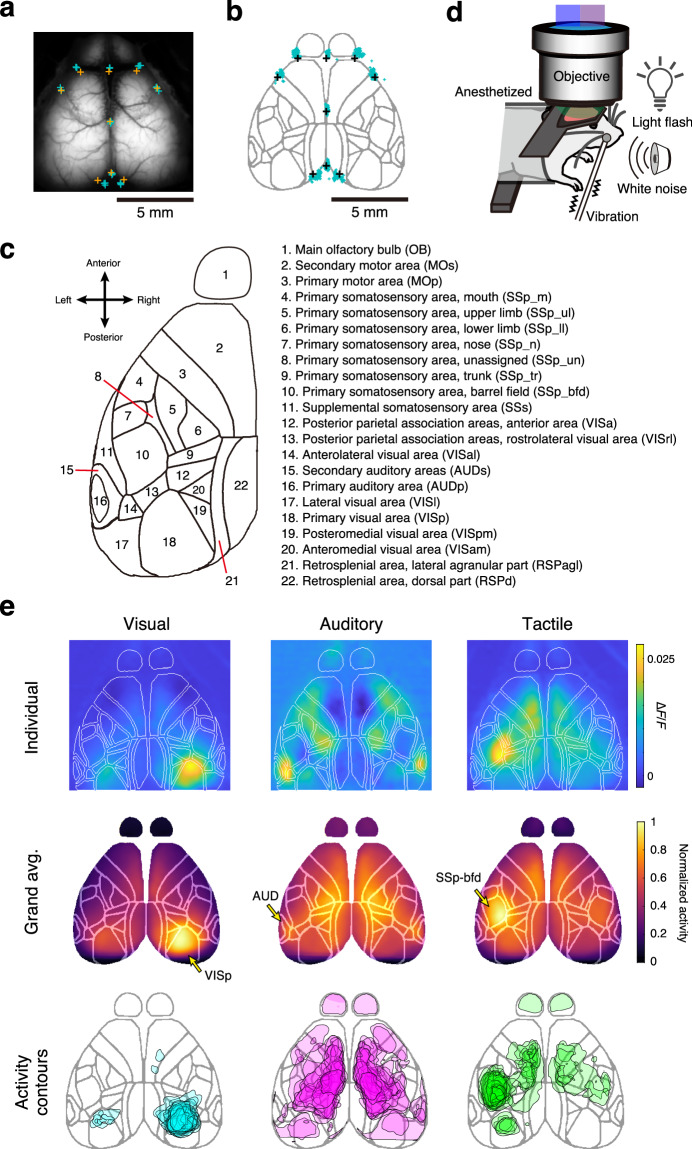
Validation of alignment to the Allen CCF reference atlas. (**a,b**) Validation of MesoNet-based skull-landmark inference. (**a**) Aligned skull landmarks from Allen CCF (orange) are plotted on the animal-average image of a representative animal and are compared with the landmarks being maƒnually annotated on three representative sessions from the animal (cyan). (**b**) Manually annotated skull landmarks are warped and plotted on the Allen CCF atlas (cyan, 75 sessions from 25 animals) and compared with the reference landmarks (black). (**c**) Coordinates and names of segmented brain regions used for the dataset, derived from the dorsal view of the Allen Common Coordinate Framework (version 3). Only the left hemisphere is shown; however, our dataset contained the ROIs of the right hemisphere as well. (**d**) Schematic illustration of the sensory-mapping experiments. Visual, auditory, and tactile stimulations were applied in the same task box as the task training sessions (see Methods for details). (**e**) Multi-modality sensory maps obtained from a representative mouse are shown (top panels). Color codes show normalized trial-averaged cortical responses (2 s duration from the stimulus onset) evoked by the three different sensory stimuli. Animal-averaged sensory maps of each modality (middle panels; *n* = 25 mice). Contours defined by more than 80^th^ percentiles in the normalized map are displayed (bottom panels). In each animal and each sensory stimulus, the evoked responses were normalized in the range from 0 to 1. Contours from 25 animals are overlaid. For this analysis, the data from the first session of the sensory-mapping experiment in each animal are used.

In providing the calcium activity from different cortical areas, Allen CCF was registered to the field of view by alignment of skull landmarks (MesoNet)^48–52^ (Fig. 2e). We validated the accuracy of this method by comparing the MesoNet-based skull-landmark annotations with the manual annotations, based on three representative sessions from each of the 25 animals (Fig. 5a,b). Through this process, cortex-wide calcium activity in each animal could be handled as ROIs in specific brain regions in a standardized brain atlas (Fig. 5c). In addition, the data from sensory-mapping sessions further validated the accuracy of our mapping (Fig. 5d,e). In these sessions, we applied visual, auditory, and tactile sensory stimulation^49,50,53^ to anesthetized mice (Fig. 5d). We obtained multimodal activity maps aligned onto the Allen CCF cortical map (Fig. 5e). Visual stimuli to the left eye of mice elicited prominent activity in the right occipital cortex^54^ corresponding to VISp (primary visual area) in the atlas. Auditory stimuli evoked spreading activity, including a multimodal sensory area in the parietal cortex^55,56^. Tactile stimuli to the right whisker pad elicited a robust response in the left SSp-bfd, a primary sensory cortex for processing whisker-related sensory information with marked hemispheric laterality^28,57^. These specific activations of the cortical area validated the accurate mapping to the Allen CCF and also confirmed that the cortical calcium signals reflected neural activity. We also confirmed that the recorded animals did not show signs of aberrant short-duration, large-amplitude calcium activity, which has been reported by others^28,57^ (Fig. S2a,b).

**Fig. 6:**
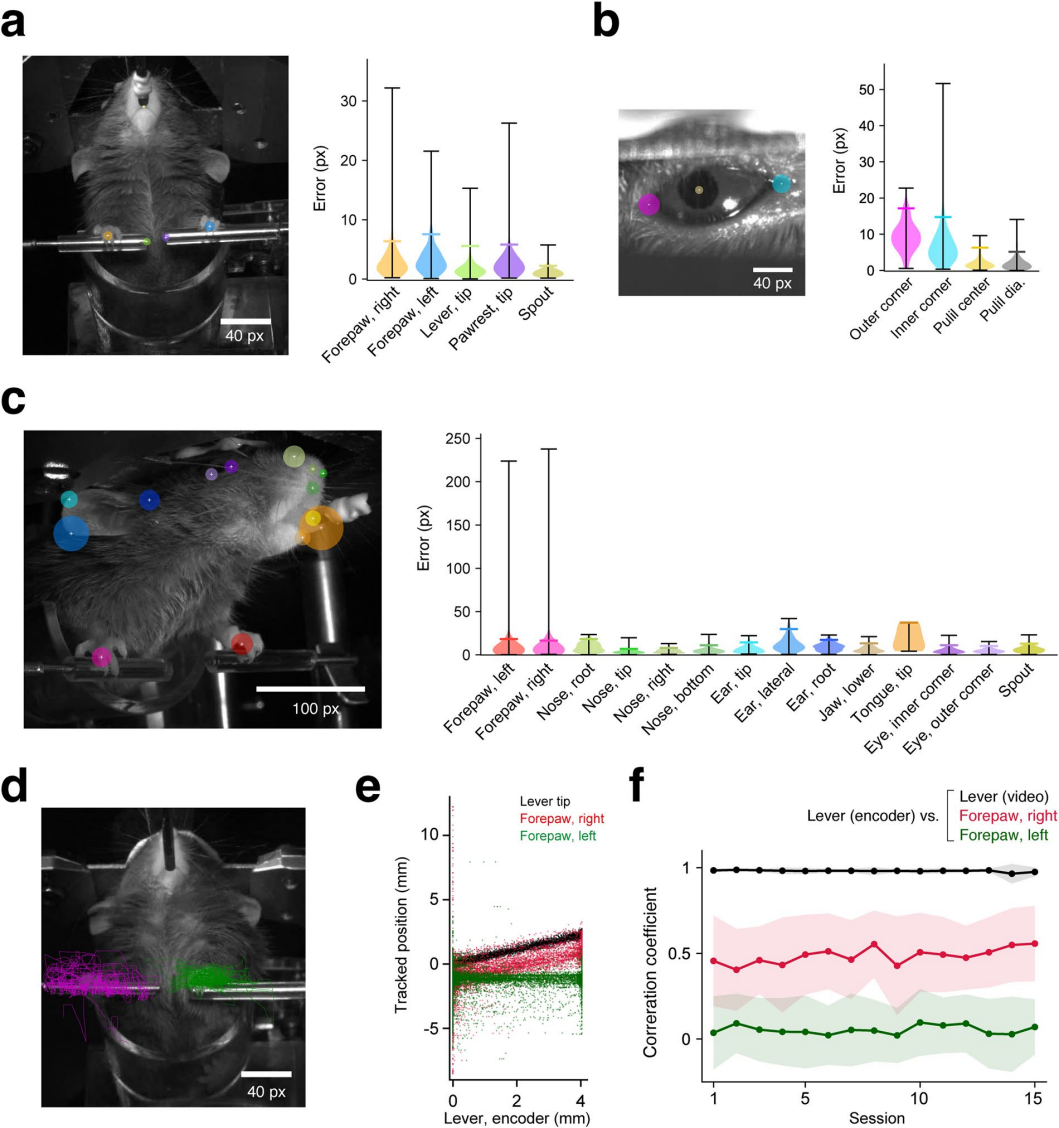
Validation of video-tracked keypoints. (**a**–**c**) DeepLabCut-inferred and manually annotated keypoints were compared using validation video frames (*n* = 192 frames from 25 animals for the body and face cameras, and *n* = 190 frames from 25 animals for the eye camera, up to 8 frames/animal; see also Table 3). In the left panels, the 95^th^ percentiles of the error distributions are indicated with colored circles overlaid on the manual annotations (white plus signs) in a representative frame of the body (**a**), right eye (**b**), and right face (**c**). The corresponding violin plots in the right panels are colored accordingly. Black whiskers indicate the minimum and maximum errors, and colored horizontal lines represent the 95^th^ percentiles. (**d,e**) Keypoint positions identified as the lever tip, the right forepaws, and the left forepaws were compared with the lever position recorded by the rotary encoder, during all successful trials of a representative session. Keypoints identified in all trials are overlaid as lines (green, left; magenta, right) on a representative frame in (**d**), and their *y*-axis positions are plotted against the encoder-based lever position in (**e**) (black, lever tip; red, right forepaw; green, left forepaw). Each dot corresponds to a single frame of the videography. (**f**) The Pearson’s correlation coefficient (*R*) between the keypoint positions and the encoder-based lever positions during successful trials was calculated across all the available task training sessions of all the animals (*n* = 25 mice, 356 sessions in total). The thick lines and shaded areas represent the mean ± s.d. across the animals.

In addition to calcium imaging data and task event timepoints, our dataset includes high-speed videography of the mice during every session. We utilized a widely used machine learning package for keypoint estimation, DeepLabCut^17,19,58–61^, to estimate the position of the body part in each frame of videos from three directions, capturing the upper body, right eye, and right side of the face (Fig. 6a–c). Comparison of DeepLabCut model-based keypoint annotations with manual annotations showed minor errors, validating the quality of our models (Fig. 6a–c). Moreover, the lever tip position estimated from the videography showed a markedly high correlation (0.98 ± 0.02; *n* = 356 sessions from 25 animals) with the encoder-based lever position (Fig. 6e,f). The position of the right forepaw used for pulling the lever should be correlated with the lever position, whereas the left forepaw should not. We found this to be exactly the case, based on the 357 task training sessions of our dataset that successfully included upper-body videos (Fig. 6d–f; right forepaw, 0.49 ± 0.23; left forepaw, 0.05 ± 0.19; *n* = 356 sessions from 25 animals; also see Table 1; one of the 357 available sessions did not contain any successful trials and was omitted from the analysis).

**Fig. 7:**
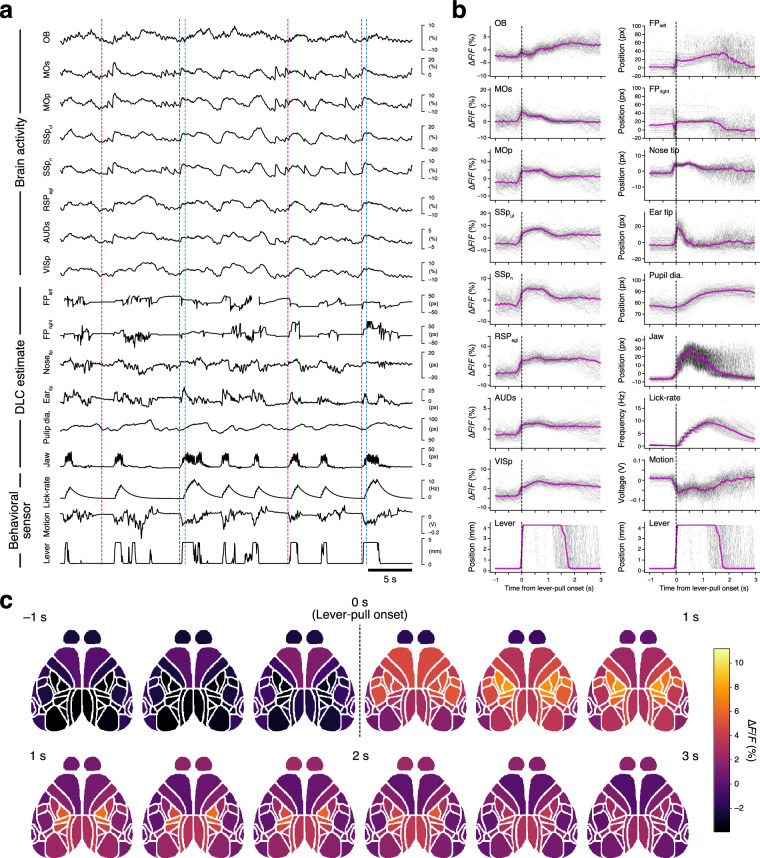
Validation of task-related behavior and activity in a representative session. (**a**) Raw trajectories of calcium dynamics from various cortical regions and video-tracked body-part movements with sensor-based behavioral records (lever movement, gross body motion, and lick rate), referenced to task events (magenta, sound cue onset; blue, reward delivery). The disconnected part in the trace of the left forepaw is caused by the value deficit with the deviation from the 5^th^ and 95^th^ percentiles of DeepLabCut (DLC) estimation. (**b**) Lever-pull-aligned traces of calcium dynamics (left column) as well as video-tracked body-part movements and sensor-based behavioral records (right column). Trial-averaged (magenta) and single-trial (black) traces from a representative session are shown. The bottom-most traces of both columns show the same lever traces as references. (**c**) Trial-averaged calcium activity was mapped onto the Allen CCF and shown in chronological order from the top left to the bottom right. Calcium activity was binned into intervals of 10 frames (333 ms), and each bin was averaged. The labels (−1 s, 1 s, 2 s, 3 s) show time relative to lever-pull onset. The data are from the same session shown in (**a,b**).

Finally, we demonstrated cortical calcium activity and video-tracked body-part movements in conjunction with sensor-based behavioral measurements for an example task training session to validate the integrity of our data (Fig. 7). In this particular session, the robust synchrony among video-tracked movements, cortical activity, and task events is apparent even in raw traces (Fig. 7a) and becomes more distinct when these signals are aligned to the lever-pull onset (Fig. 7b)^17,19,58–61^. Certain body parts, such as forepaws and nose, began moving before lever-pull onset, whereas others, including jaw and pupil diameter, moved afterward, at least in this session. Aligned traces of many cortical areas show onsets similar to lever-pull movements, and motor and somatosensory areas showed that the activity relatively correlated with lever-pull movements. We found slight differences between them. The secondary motor cortex (MOs) quickly recovered to baseline level. By contrast, the primary motor (MOp) and somatosensory cortices (SSpul and SSpn) showed activity that was slightly higher than the baseline level after the reward delivery. Other sensory-related areas (OB, olfactory bulb; RSPagl, retrosplenial area, lateral agranular part; AUDs, secondary auditory areas; VISp, primary visual area) showed activity traces that shared time courses closely matching pupil dilation. The data also revealed a decrease in activity variability after the lever-pull onset, exemplifying how stereotypic movements, such as the lever pull, constrain cortical activity. Furthermore, our cortical activity data inherently contains spatial information and has been preprocessed to a ready-to-use format, and thus the spatiotemporal dynamics of cortical activity are readily accessible (Fig. 7c).

## Supplementary information

Supporitng Information

## Data Availability

All workflows presented in this paper were implemented in MATLAB and Python, and the source code is publicly available (MIT License) at https://github.com/BraiDyn-BC/bdbc-data-pipeline.
