# Supplementary material for: Multimodal dataset linking wide‐field calcium imaging to behavior changes in operant lever‐pull task in mice
Source: Sci Data. 2025 Jul 29;12:1264. doi: 10.1038/s41597-025-05482-y (PMC12307678; doi:10.1038/s41597-025-05482-y)
Supplement: Supplementary file 1 — Supporitng Information [file 41597_2025_5482_MOESM1_ESM.docx]

| **Software** | **Version** | **Usage** |
| --- | --- | --- |
| HCImage | 5.0.2.2 | Control sCMOS camera and image acquisition for one-photon calcium imaging |
| Labview | 2021 | Control behavioral devices and recordings |
| Pylon viewer | 7.2.1.25747 | Control machine vision cameras and video acquisition |
| MATLAB | 2022b | Behavioral data processing (filtering, event detection, lick-rate calculation, temporal down-sampling to synchronize for calcium imaging data) |
| NoRMCorre | 0.1.1 | Motion correction for calcium image stacks |
| Python | 3.10.14 | Data processing pipeline for packaging into NWB files |
| Conda | 24.3.0 | Python environment management |
| NumPy | 1.24.3 | Numeric array handling |
| SciPy | 1.14.0 | Δ*F*/*F* signal processing |
| Scikit-learn | 1.5.1 | Δ*F*/*F* signal processing |
| OpenCV (python binding) | 4.10.0 | Image processing |
| Pandas | 2.2.2 | Data storage |
| h5py | 3.11.0 | Data storage |
| imageio | 2.34.2 | Reading from and writing to images |
| Tensorflow | 2.12.1 | Training and running of deep neural networks |
| DeepLabCut | 2.3.10 | Keypoint estimation from behavior videos |
| imgaug | 0.4.0 | Data augmentation for training DeepLabCut models |
| ks-pupilfitting(*) | 0.1.2 | Pupil tracking based on the results of DeepLabCut inference |
| bdbc-atlas-registration(*) | 0.2.0 | Alignment of the reference atlas to calcium imaging data |
| ks-mesoscaler(*) | 0.1.0 | Alignment of the reference atlas to calcium imaging data |
| ks-affine-aligner(*) | 0.1.0 | Alignment of calcium imaging data across sessions of the same animal |
| ks-affine2d(*) | 0.1.0 | Procedures related to affine transformation |
| PyNWB | 2.8.1 | NWB file handling |
| bdbc-nwb-packager(*) | 0.5.1 | Packaging data into NWB files |

**Table S1** | Version and usage information of representative software and libraries. *These libraries are part of our code repository.

**
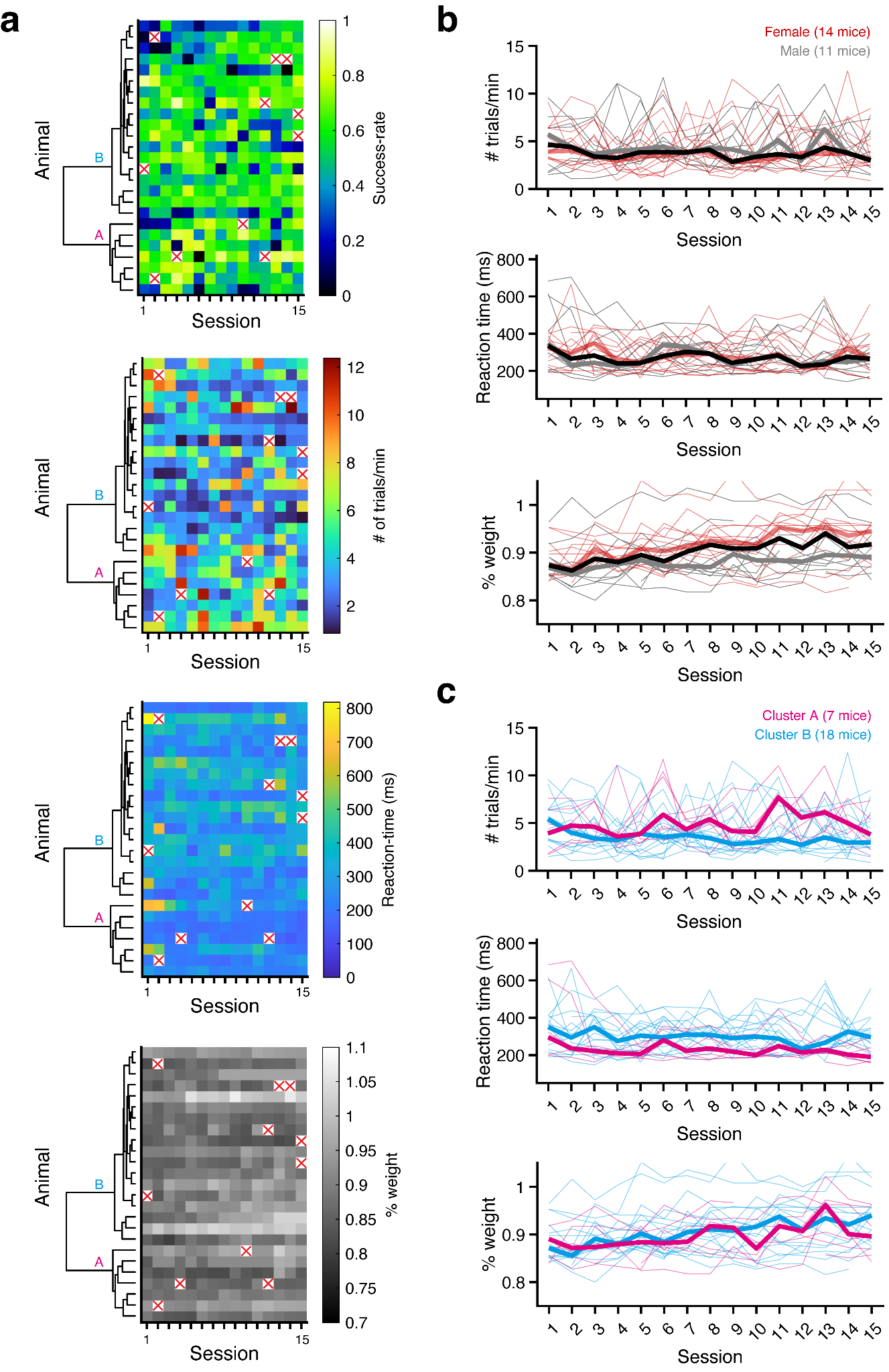
**

**Figure S1 |** Behavioral performance in task training sessions. **a**, Four different obtained behavioral parameters are color-coded. **b**, The trial number/min (top panel), reaction time in all lever-pulled trials (middle panel), and body weight normalized to the weight before starting water restriction (bottom panel) across sessions. Thin lines denote individual animals (red, female; gray, male), whereas thick lines denote the median across the population (red, female; gray, male; black, all). **c**, Behavioral parameters of individuals in clusters A (magenta; *n* = 7) and B (cyan; *n* = 18), identified in Fig. 3d. These plots are shown similarly as in (**b**).

**
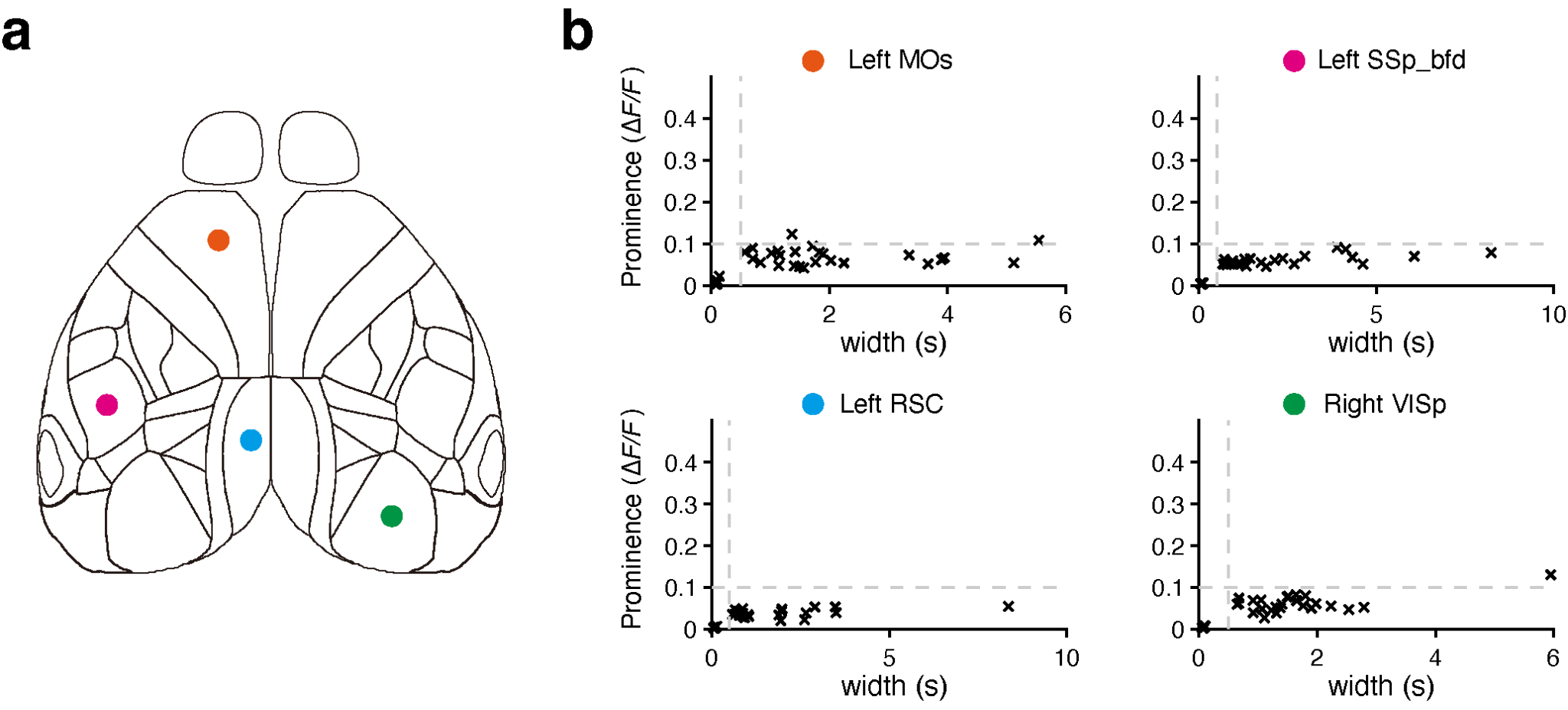
Figure S2 |** The validation of aberrant activity in the transgenic mouse was used in this study. **a**, The following positions for considering aberrant activities were selected for analyses: the left side of the frontal, barrel, retrosplenial, and right side of visual regions. **b**, Prominences and widths of calcium activities were detected in each first resting-state recording of each animal. These were clustered with K-means clustering (k = 2) in each animal and region, and then the centers of mass of two clusters were obtained. High prominence (> 0.1 in ∆*F*/*F*) and narrow width (< 0.5 s) of the transients were not detected in each animal.


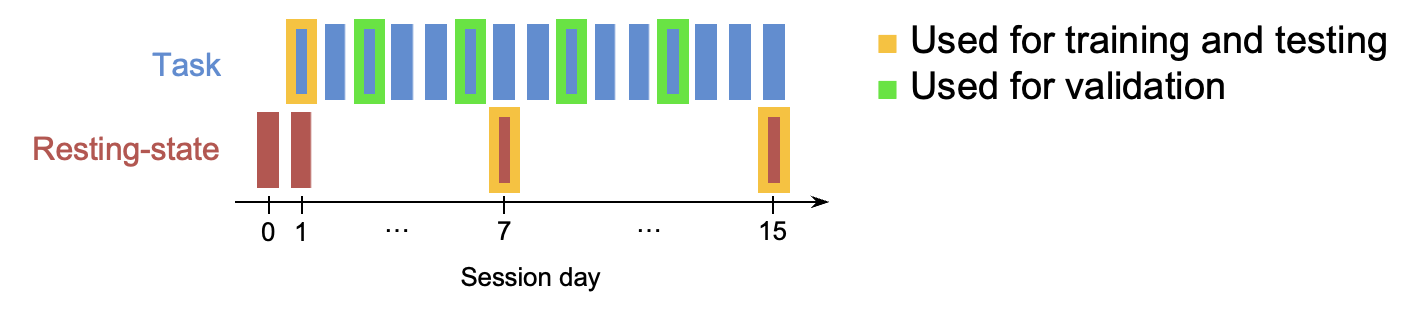


**Figure S3** | Schematic of frame extraction strategy for training and validating DeepLabCut models. Task training session 1 and resting-state recording sessions 7 and 15 (including the day-8 resting-state session from one animal) were used to extract video frames for training and testing. Validation frames, for comparison with manual annotation, were extracted from task training sessions 3, 6, 9, and 12.


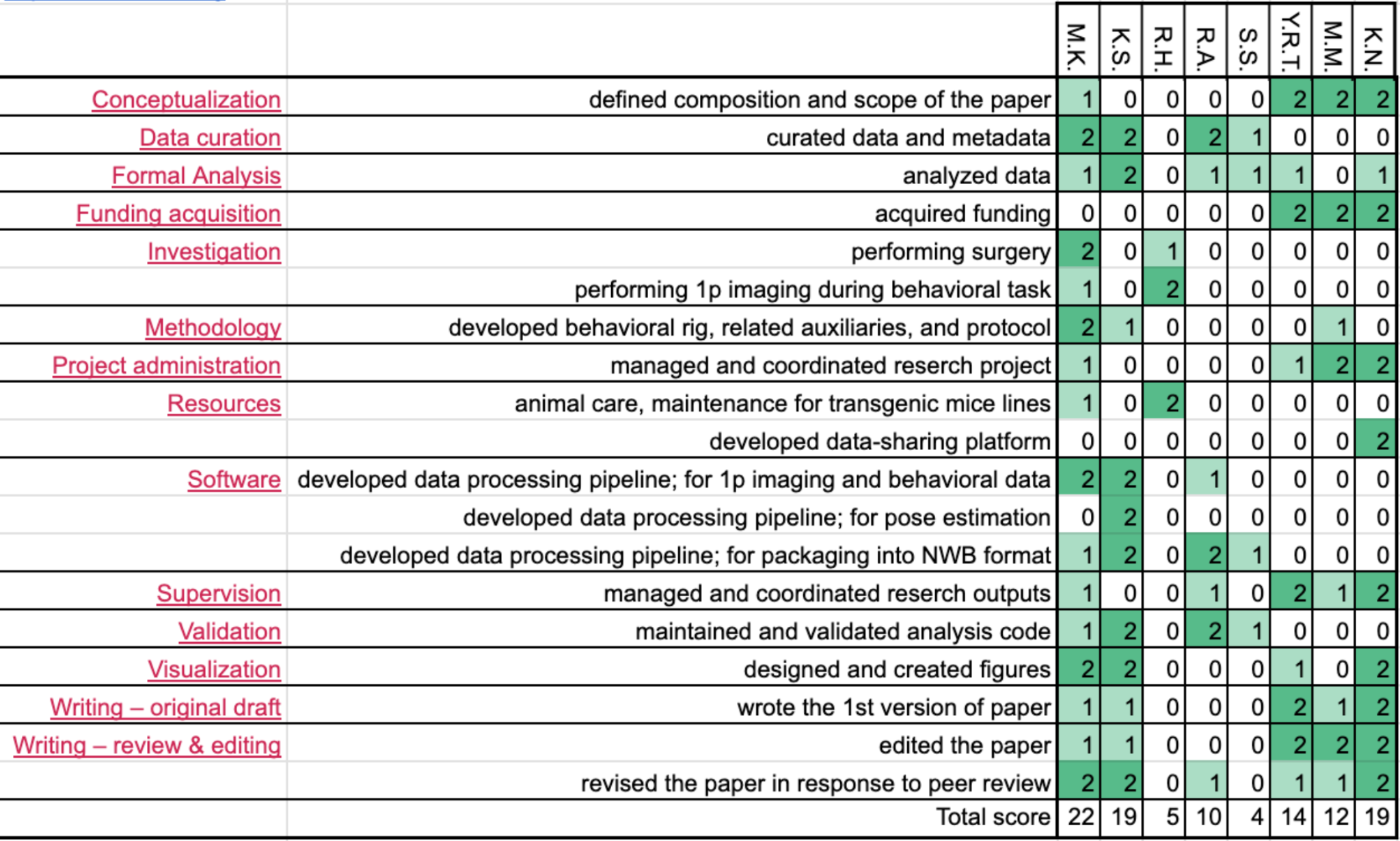


**Figure S4** | Contribution diagram. The following diagram illustrates the contributions of each author, based on CRediT taxonomy (Brand et al., 2015). For each type of contribution, two levels are color-indicated: ‘support’ (light green) and ‘lead’ (dark green).
